# Supplementary material for: Effects of Freshwater Acidification on the Gut Microbial Community of Trachemys scripta elegans
Source: Animals (Basel). 2024 Jun 27;14(13):1898. doi: 10.3390/ani14131898 (PMC11240511; doi:10.3390/ani14131898)
Supplement: Supplementary file 1 [file animals-14-01898-s001.zip › Supplementary File.pdf]

Supplementary File S1. Number and length sequences in the samples.

Sample\Info	Seq_num	Base_num	Mean_length	Min_length	Max_length
pH5.5_1	42172	17578120	416.82	271	430
pH5.5_2	50721	20997234	413.98	250	442
pH5.5_3	54581	22292506	408.43	217	432
pH5.5_4	57747	23850136	413.01	230	431
pH5.5_5	48451	19862816	409.96	222	430
pH5.5_6	42950	17636219	410.62	255	431
pH5.5_7	49830	20312495	407.64	259	431
pH5.5_8	48263	19870134	411.71	223	431
pH5.5_9	45622	18639631	408.57	301	450
pH6.5_1	50298	20373755	405.06	221	501
pH6.5_2	49298	20427370	414.37	265	431
pH6.5_3	41451	16931780	408.48	233	432
pH6.5_4	49009	20101954	410.17	325	430
pH6.5_5	46257	19072400	412.31	317	463
pH6.5_6	46738	19168394	410.12	221	433
pH6.5_7	51909	21382301	411.92	258	431
pH6.5_8	45569	18781959	412.17	251	432
pH6.5_9	42149	17502570	415.25	219	434
pH6.5_10	46825	19273441	411.61	302	431
pH7.5_1	41633	17067806	409.96	302	431
pH7.5_2	42271	17584835	416.00	204	434
pH7.5_3	42745	17487204	409.11	212	524
pH7.5_4	47303	19332514	408.70	251	431
pH7.5_5	45449	18550984	408.17	272	482
pH7.5_6	45598	18784415	411.96	255	432
pH7.5_7	45483	18617026	409.32	232	473
pH7.5_8	47604	19526360	410.18	250	435
pH7.5_9	64441	26351990	408.93	258	431
pH7.5_10	47418	19473453	410.68	277	431

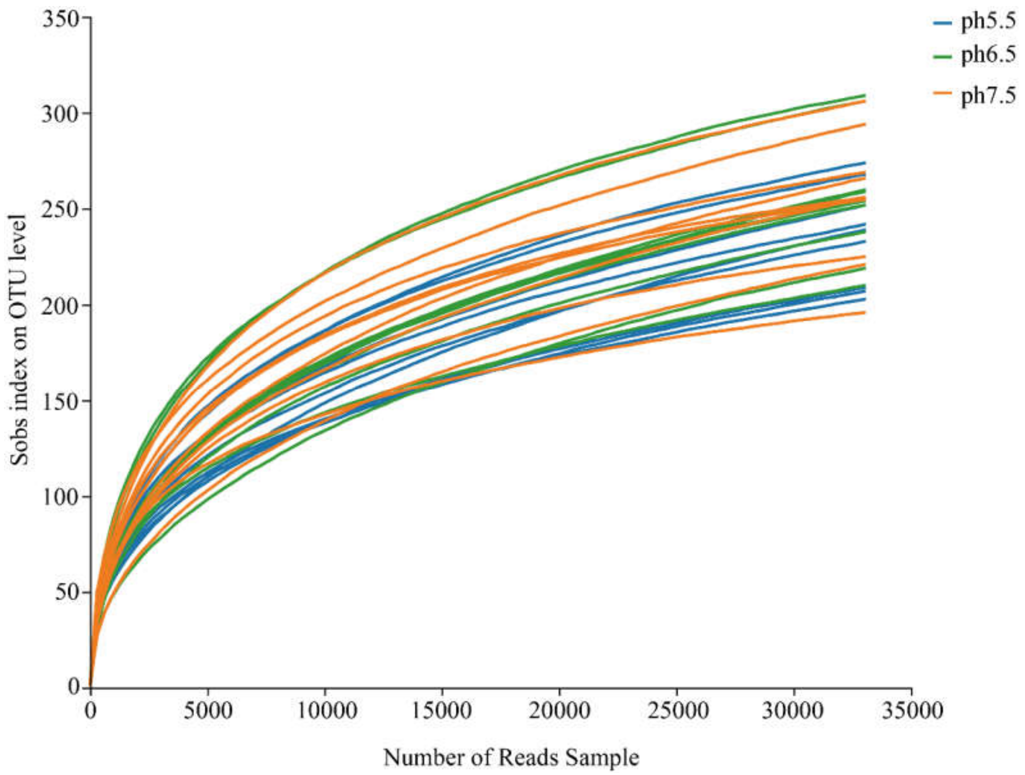

Supplementary File S2. Sequencing dilution curves of sample.
